# Resilient Hearts: A Journey of Battling Endemic Diseases in Lyari Town

**DOI:** 10.4269/ajtmh.23-0398

**Published:** 2023-11-20

**Authors:** Raja Ram Khenhrani

**Affiliations:** Department of Internal Medicine, Lyari General Hospital, Karachi, Pakistan

When I started my residency, I had no idea that I would spend nearly 6 months of my precious time venturing through Lyari Town, but the moment I walked into the emergency department on my very first day of residency, my life changed. A young boy grabbed my arm, imploring me to save his grandmother’s life. I followed him to the corridor, where I found an elderly woman who appeared cachectic and lethargic. She was lying on a stretcher with deeply sunken eyes, a pale face, and chapped lips—all telltale signs of severe dehydration that I had learned in medical school. I promptly inserted a wide-bore intravenous cannula into her left arm, pushed saline boluses, and transferred her to the high-dependency unit, where I continued to oversee her care.

As I started my initial rotation in the high-dependency unit, I saw that the young boy stayed by his grandmother’s side. As days turned into a week, the elderly woman’s eyes regained their sparkle, her skin became less pale, and her lips transformed into a grateful smile. I bid farewell to her on my last day in the unit, leaving her with instructions on food hygiene. This was my first encounter with cholera during my residency, but she was not the only patient from Lyari Town to enter Lyari General Hospital with a preventable infectious disease.

During my very first week on the hospital floors, I encountered a total of 72 cases of preventable diarrheal illnesses, all related to enteric fever, infectious colitis, and giardiasis. This included my first death.

Even now, when I close my eyes, I picture the patient’s grieving husband standing in the hallway. Tears streaming down his face, he pulled out a crumpled bunch of antibiotic prescriptions from his pocket and looked at them in anguish—a desperate attempt to show me the multitude of medications his wife had taken during her battle. But none of the antibiotics worked against the extremely drug-resistant typhoid that killed her through septicemia.

After seeing this suffering, I needed to do something to prevent it. Along with four other internal medicine residents, we began to volunteer in Lyari Town. We went door to door, house to house. On that first day, we did not have a clear plan. I found myself sitting on a charpais (a handcrafted cot) in a small house with a leaking roof and missing windows talking about typhoid when a 14-year-old girl pulled up her long, embroidered tunic to reveal a scar on her abdomen and inquired “do you know how I got this scar?”

Curious, I replied, “No,” and listened intently.

“This scar is the result of the surgery I underwent due to typhoid.” She further asked, “Do you know how I acquired typhoid?” I again carefully listened as she replied, “Unpurified water!”

It turns out that water taps in Lyari Town dispense unpurified water for most of the day. Even though purified water is accessible for a limited period, from midnight to 2:00 am, its quality is compromised because of dirty pipes. We learned this from a pleasant middle-aged lady with a fair complexion, almond-shaped eyes, and a melodious voice. “Why just 2 hours?” I inquired.

She explained, “Lyari’s electricity gets cut off for nearly 18 hours every day.” She further advised, “why don’t you create informative pamphlets tailored to the specific needs of Lyari Town?” It was our first day of volunteering in Lyari Town, and little did I know that it would lead us on an incredible 6-month journey, visiting all 700+ houses in Lyari Town.

As the second day of our volunteering began, each of us created educational paper pamphlets. Armed with our pamphlets, we opted to ride into Lyari Town on rickshaws, each driven by a seasoned local driver. The sights on the streets were less than idyllic—small heaps of rubbish scattered around street corners, open sewers brimming with waste, and stagnant pools of water mirroring the fractured roads. Despite the obstacles, we tirelessly distributed pamphlets to every possible house on the street. Moreover, we personally engaged with the members of each household, listened to their concerns, and taught them about certain practices: boiling undrinkable water, washing hands with soap and water for at least 30 seconds before every meal, and availing themselves of the vaccination opportunity against typhoid offered by Lyari General Hospital.

Day after day, our efforts persisted against diarrheal disease, and the months began to roll by. Soon we had completed our visits to every possible house in Lyari Town. Several questions flooded my mind at the end of this journey. How many families in town would follow our advice? Did the townspeople understand the diseases that are common in their area? Will the people of Lyari Town understand the importance of getting vaccinated against typhoid? Will the residents engage in disease prevention and control efforts?

Within a month, I observed that our efforts were beginning to bear fruit. While volunteering for the typhoid vaccination program at Lyari General Hospital, I encountered the same child who had once pleaded with me to save his grandmother’s life. Our eyes met, and a smile instantly lit up both our faces. He had grown in stature, radiated a pleasant personality, and had curly hair and a beautifully tanned complexion. With a gleam of recognition in his eyes, he came forward and said, “Sir, I have been spreading your words about drinking purified water, the advice that you gave to my grandmother when she was discharged from the high-dependency unit.”

In another encounter, the husband who had lost his wife from extremely drug-resistant typhoid approached me. With gratitude in his eyes, he admired our efforts, saying, “Doctor, you are doing an amazing job. Do you know what role I am playing to protect my two daughters and son from typhoid?” I looked at him with curiosity, waiting for his response. Proudly, he said, “I am getting them vaccinated against typhoid today, just as written on your pamphlets.” These words filled me with a profound sense of reassurance, reaffirming the impact of our ongoing efforts in the fight against infectious yet preventable diseases.

